# PPARδ-mediated mitochondrial rewiring of osteoblasts determines bone mass

**DOI:** 10.1038/s41598-020-65305-5

**Published:** 2020-05-21

**Authors:** Dorothea I. H. Müller, Cornelia Stoll, Katrin Palumbo-Zerr, Christina Böhm, Brenda Krishnacoumar, Natacha Ipseiz, Jule Taubmann, Max Zimmermann, Martin Böttcher, Dimitrios Mougiakakos, Jan Tuckermann, Farida Djouad, Georg Schett, Carina Scholtysek, Gerhard Krönke

**Affiliations:** 1 Department of Internal Medicine 3, Rheumatology and Immunology, Friedrich Alexander University Erlangen-Nurnberg and Universitätsklinikum Erlangen, Erlangen, Germany; 2 Nikolaus Fiebiger Center of Molecular Medicine, University of Erlangen- Nuremberg, Erlangen, Germany; 3 Systems Immunity Research Institute, Heath Park, Cardiff University, Cardiff, United Kingdom; 4 Department of Internal Medicine 5, Hematology and Oncology, Friedrich Alexander University Erlangen-Nurnberg and Universitätsklinikum Erlangen, Erlangen, Germany; 5 Institute of Comparative Molecular Endocrinology, University of Ulm, Ulm, Germany; 6 IRMB, University Montpellier, INSERM, Montpellier, France

**Keywords:** Cell biology, Drug discovery, Bone, Bone quality and biomechanics, Metabolism

## Abstract

Bone turnover, which is determined by osteoclast-mediated bone resorption and osteoblast-mediated bone formation, represents a highly energy consuming process. The metabolic requirements of osteoblast differentiation and mineralization, both essential for regular bone formation, however, remain incompletely understood. Here we identify the nuclear receptor peroxisome proliferator-activated receptor (PPAR) δ as key regulator of osteoblast metabolism. Induction of PPARδ was essential for the metabolic adaption and increased rate in mitochondrial respiration necessary for the differentiation and mineralization of osteoblasts. Osteoblast-specific deletion of PPARδ in mice, in turn, resulted in an altered energy homeostasis of osteoblasts, impaired mineralization and reduced bone mass. These data show that PPARδ acts as key regulator of osteoblast metabolism and highlight the relevance of cellular metabolic rewiring during osteoblast-mediated bone formation and bone-turnover.

## Introduction

Bone formation and bone remodeling represent highly energy demanding processes^1^. Especially the unique vascular architecture of long bones as well as large gradients in oxygen tension and nutrient supply impose metabolic challenges for bone resident cells^2,3^. Insights into the mechanisms that allow osteoblasts and osteoclasts the metabolic adaption to their specific microenvironment are therefore essential to understand both physiological and pathological bone turnover. Osteoblasts in long bones can sense low oxygen tension leading to a stabilization of hypoxia-induced factor (HIF) proteins, a process that controls both VEGF-dependent vascularization of bones and erythropoiesis within the bone marrow^4,5^. On a cellular level, HIF-1alpha-driven glycolysis in osteoblasts also directly contributes to bone formation^6^ and bone anabolic factors such as agonists of the Wnt signaling pathway can stimulate aerobic glycolysis in osteoblasts^7^. However, osteoblasts were also shown to consume and utilize large amounts of fatty acids^8,9^. In accordance, osteogenic differentiation of mesenchymal stem cells (MSCs) was reported to be paralleled by an increased mitochondrial respiration, where levels of ATP derived from oxidative phosphorylation peak in association with the accumulation of mitochondria that can directly support differentiation of osteoblasts^10,11^.

These findings show that especially differentiating and mineralizing osteoblasts utilize both aerobic glycolysis and oxidative phosphorylation, indicating that these cells need to adjust their bioenergetic machinery in order to adapt to transient metabolic challenges such as low and high oxygen and varying nutrient supply. The exact reason for the usage of divergent energy pathways as well as the *in vivo* relevance of the observed increase in oxidative phosphorylation in differentiating osteoblasts remains unclear. Notably, glucose transporter 1 (Glut1)-mediated glucose uptake has been recognized as an important signal that initiates early osteoblast commitment by regulating the stability of Runt-related transcription factor 2 (Runx2)^12^. The signals that control the induction of mitochondrial respiration dominating during later stages of osteoblast differentiation and mineralization, in turn, have remained elusive.

## Results

### Osteoblast differentiation is dependent on an increase in oxidative phosphorylation

**Figure 1:**
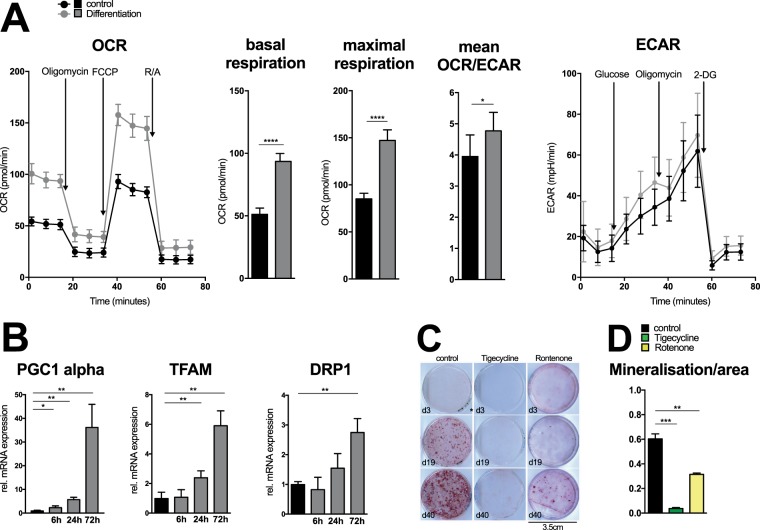
Osteoblast differentiation is dependent on an increase in oxidative phosphorylation. **(A)** Oxygen consumption rate (OCR) and the extracellular acidification rate (ECAR) including basal and maximal respiration rate measured with extracellular flux (XF) analyzer in freshly isolated calvarial osteoblasts cultured in regular growth medium (black) and differentiation medium (grey) for 24 hours (n = 9 each). **(B)** Real-time PCR analysis of mRNA expressions normalized to β-actin in calvarial osteoblasts cultured in regular growth media (black) and differentiation media (grey) for 6, 24 and 72 hours (n = 3). **(C)** Alizarine Red staining of calvarial osteoblasts cultured in osteoblastic differentiation media supplemented with 10 ng/ml Wnt3a and conditionally supplemented with vehicle (control), 30 µM tigecycline or 20 nM Rotenone at day 3, 19 and 40 of culture (representative for n = 3). **(D)** Quantitative analysis of mineralized areas of alizarine red staining of calvarial osteoblasts supplemented with 10 ng/ml Wnt3a and conditionally supplemented with 30 µM Tigezycline (green) or 20 nM Rotenone (yellow) cultured in osteoblastic differentiation media for 40 days (n = 3). *P ≤ 0.05, **P ≤ 0.01, ***P ≤ 0.005 using two-tailed Student’s t-test.

To determine the metabolic requirements for regular osteoblast differentiation, we initially performed an extracellular flux analysis of in vitro cultured primary osteoblasts and their precursors. We compared the metabolic profiles of osteoblast precursors during steady state and upon initiation of osteoblast differentiation. These experiments confirmed a significantly increased oxygen consumption and oxidative phosphorylation of differentiating osteoblasts (Fig. 1A). Although the glycolytic activity slightly increased as well, this metabolic adaption resulted in an increase in the ratio between oxygen consumption rate (OCR, an indicator for mitochondrial respiration) and the extracellular acidification rate (ECAR, an indicator for glycolysis), suggestive of a robust metabolic rewiring of differentiating osteoblasts. In accordance, we observed a time-dependent increase in the expression of multiple genes involved in the control of mitochondrial respiration, mitochondrial biogenesis and oxygen-dependent energy provision such as Peroxisome proliferator-activated receptor gamma coactivator 1 (PGC1)α, mitochondrial transcription factor A (TFAM) or dynamin-1-like protein (DRP)1 in differentiating osteoblasts (Fig. 1B). Pharmacologic inhibition of mitochondrial biogenesis by tigecycline^13^ or of oxidative phosphorylation by rotenone, in turn, did not interfere with osteoblast viability, but dramatically diminished their differentiation and mineralization potential (Fig. 1C,D and Suppl. Fig. 1). These data indicated a global shift in the transcriptional program that controlled the cellular metabolic adaption during osteoblast differentiation and mineralization.

### The nuclear receptor PPARδ mediates metabolic rewiring of osteoblasts

**Figure 2:**
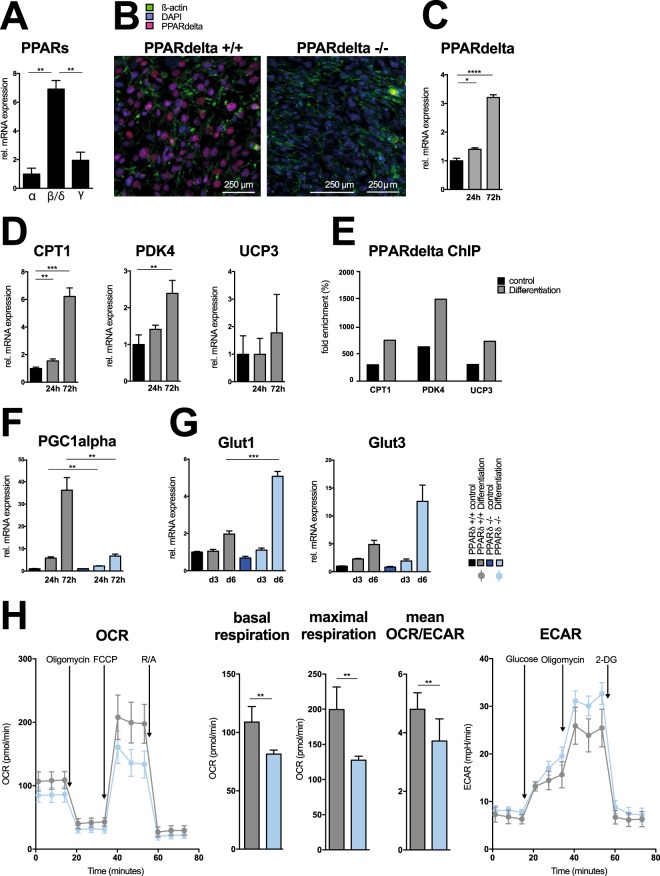
PPARδ-mediated metabolic rewiring of osteoblasts. **(A)** Quantitative real-time PCR analysis of PPAR isoforms (α, δ and γ) normalized to β-actin in calvarial osteoblasts cultured in differentiation media for 24 hours (n = 3). **(B)** Immunofluorescence microscopy of PPARδ wildtype and PPARδ-deficient MSCs cultured in differentiation medium for 7 days (β-actin, green; DAPI, blue; PPARδ, red; representative for 3 independent experiments). **(C)** Quantitative real-time PCR analysis of PPARδ mRNA expression normalized to β-actin of osteoblasts cultured in regular growth medium (black) and differentiation media (grey) for 24 and 72 hours (n = 3). **(D)** Real-time PCR analysis of mRNA expressions of CPT, PDK4 and UCP3 normalized to β-actin in calvarial osteoblasts cultured in regular growth media (black) and differentiation media (grey) for 24 and 72 hours (n = 3). (E) Chromatin immunoprecipitation (ChIP) assays determining binding of PPARδ to the promoters of indicated genes in calvarial osteoblasts cultured in regular growth media (black) and differentiation media (grey) for 72 hours. (**F**,**G)** Real-time PCR analysis of mRNA expressions of **(F)** PGC1alpha and **(G)** Glut1 and Glut3 normalized to β-actin in PPARδ wildtype (black and grey) and PPARδ-deficient (dark and light blue) calvarial osteoblasts cultured in either regular growth media or differentiation media (n = 3). **(H)** OCR, ECAR, basal and maximal respiration rate measured in freshly isolated PPARδ wildtype (grey) and PPARδ-deficient (light blue) calvarial osteoblasts cultured in differentiation medium for 24 hours (n = 3). *P ≤ 0.05, **P ≤ 0.01, ***P ≤ 0.005, **** P ≤ 0.001 using two-tailed Student’s t-test.

PGC1α is considered a master regulator of mitochondrial biogenesis and was among the most highly induced genes in differentiating osteoblasts. As PGC1alpha serves as co-activator and interacts with various other transcription factors, we next assessed expression levels of genes encoding for proteins that represent known PGC1α interaction partners involved in cellular metabolism and oxidative phosphorylation. This analysis identified peroxisome proliferator-activated receptor (PPAR)δ as one of the prominently expressed transcription factors in differentiating osteoblasts (Fig. 2A–C). PPARδ belongs to the superfamily of nuclear receptors and acts as a ligand-dependent transcription factor that senses fatty acids and subsequently controls fatty acid oxidation, a process that primarily fuels mitochondrial respiration. We have previously identified PPARδ as important regulator of Wnt signaling and osteoblast/osteoclast crosstalk^14,15^. Our current analysis showed that expression of PPARδ gradually increased during early osteogenic differentiation, whereas expression levels of its family members PPARα and PPARγ remained low (Fig. 2A–C). We additionally confirmed expression of PPARδ on a protein level, which was induced during osteogenic differentiating of wild-type, but not in PPARδ-deficient MSCs (Fig. 2B). In accordance, induction of PPARδ was paralleled by induction of several known PPARδ target genes such as carnitine palmitoyltransferase (CPT)-1 or pyruvate dehydrogenase kinase (PDK)4, with expression levels likewise increasing during osteoblast differentiation (Fig. 2D). As expected, chromatin immunoprecipitation experiments confirmed direct binding of PPARδ to these known target genes also in osteoblasts (Fig. 2E). Next, we sought to determine whether induction of PPARδ indeed controlled the metabolic rewiring and increase in oxidative phosphorylation we had observed during osteogenic differentiation. We consequently analyzed the metabolic phenotype of PPARδ-deficient osteoblasts. Absence of PPARδ resulted in suppression of the expression of metabolic key genes such as PGC1α (Fig. 2F). This finding indicated that PPARδ controlled a transcriptional program that regulated mitochondrial metabolism and oxidative phosphorylation during osteoblast differentiation. The expression levels of metabolic genes such as glucose transporter (Glut)1 or Glut3, which are involved in glycolysis, in turn, were not suppressed, but even increased in PPARδ-deficient osteoblasts (Fig. 2G). The PPARδ-mediated regulation of genes involved in oxidative phosphorylation accordingly resulted in a reduced oxygen consumption and impaired oxidative phosphorylation in PPARδ-deficient osteoblasts, whereas glycolysis was increased (Fig. 2H).

### Absence of PPARδ in osteoblasts alters their differentiation and mineralization

**Figure 3:**
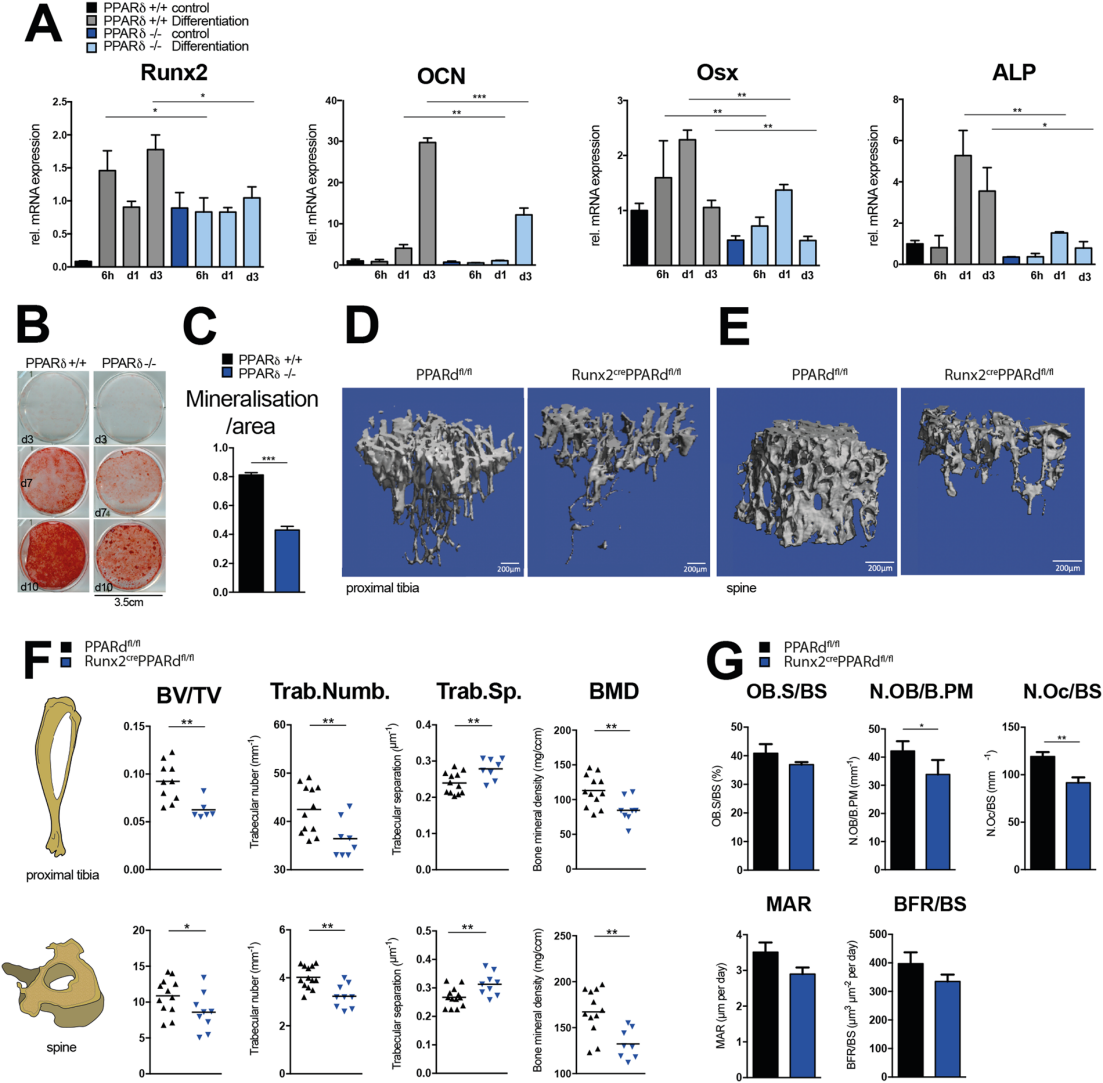
Absence of PPARδ in osteoblasts alters their differentiation and mineralization. **(A)** Real-time PCR analysis of mRNA expressions of Runx2, OCN, Osx and ALP normalized to β-actin in PPARδ wildtype (black and grey) and PPARδ-deficient (dark and light blue) calvarial osteoblasts cultured in either regular growth medium or differentiation media for 6, 24 and 72 hours (n = 3). **(B)** Alizarine Red staining of PPARδ wildtype and PPARδ-deficient MSCs cultured in osteoblastic differentiation media supplemented with 10 ng/ml Wnt3a at day 3, 7 and 10 of culture (n = 3). **(C)** Quantitative analysis of mineralized areas of alizarine red staining of PPARδ wildtype (black) and PPARδ-deficient (blue) MSCs cultured in osteoblastic differentiation medium for 10 days (n = 3). **(D,E)** µCT images of **(D)** tibial and **(E)** vertebral bone of female wild type mice (*PPARd*^fl/fl^) or their female littermates carrying an osteoblast-specific deletion of PPARδ (*Runx2*^cre^*PPARd*^fl/fl^) at 12 weeks of age. **(F)** µCT-based analysis of structural bone parameters including bone volume per total volume (BV/TV), trabecular number (Trab.Numb.), trabecular separation (Trab.Sp.) and bone mineral density (BMD) of tibial (upper panel) and vertebral (lower panel) bone of female wild type mice (black) or their female littermates carrying an osteoblast-specific deletion of PPARδ (blue) at 12 weeks of age (n = 6–9). µCT analysis was performed using the operating system “Open VMS” by SCANCO Medical (Version 6.1). **(G)** Histomorphometric analysis of percentage of surfaces covered by osteoblast (OB.S/BS), osteoblast number (N.OB/B.PM), osteoclast number (N.Oc/B B.PS), mineral apposition rate (MAR) and ratio of bone formation to bone surface (BFR/BS) counted in the proximal tibia of wild type mice (black) or their female littermates carrying an osteoblast-specific deletion of PPARδ (blue) at 12 weeks of age (n = 3). *P ≤ 0.05, **P ≤ 0.01, ***P ≤ 0.005 using two-tailed Student’s t-test.

To understand whether osteoblast differentiation and/or mineralization were dependent on this PPARδ-mediated metabolic rewiring, we studied osteoblast differentiation in the absence of PPARδ. In comparison to wild-type osteoblast, PPARδ-deficient cells showed a defective upregulation of osteoblast differentiation markers such as Runx2, osteocalcin (OCN), osterix (Osx), and alkaline phosphatase (ALP) (Fig. 3A). PPARδ-deficient MSCs accordingly displayed a defective mineralization upon induction of osteogenic differentiation, demonstrating an important intrinsic role of the PPARδ-controlled metabolic adaption during osteoblast differentiation and function (Fig. 3B,C). To determine the *in vivo* relevance of the observed phenotype, we generated mice carrying an osteoblast-specific deletion of PPARδ. Analysis of these *Runx2*^cre^*PPARd*^fl/fl^ mice showed that these animals displayed a reduced bone mass in comparison to their wild type littermates with a significantly decreased bone volume/total volume, lower trabecular number and decreased bone mineral density in both tibia and spine (Fig. 3D–F). This low bone mass phenotype was associated with a reduced number of osteoblasts and a tendency towards a reduced mineral apposition and bone formation rate (Fig. 3G).

## Discussion

Nuclear receptors including the members of the PPAR family control pleotropic processes throughout the body, including control of fatty acid and glucose metabolism in liver, adipose tissue and muscle^16^. Previous data have identified PPARδ as important regulator of bone turnover and musculoskeletal homeostasis^14,17^. Although these studies determined a contribution of this nuclear receptor during muscle and bone biology as well as during osteoblast/osteoclast crosstalk, its exact molecular role during bone formation remained unclear. Our current data expand these insights on the involvement of this nuclear receptor in osteoblast biology and reveal an osteoblast-intrinsic role of this nuclear receptor during bone homeostasis *in vivo*. We show that PPARδ regulates the metabolic adaption and increased mitochondrial respiration, events that are required for efficient osteoblast differentiation and mineralization.

It remains to be determined to which extend the PPARδ-mediated control of energy homeostasis accounts for the reduced mineralization and bone mass observed in *Runx2*^cre^*PPARd*^fl/fl^ mice and whether additional effects exerted by this transcription factor contribute to this phenotype. Control of osteoblast-intrinsic mitochondrial respiration, however, seems to critically influence differentiation and mineralization of this cell type. Although mice that carry a Sox2-mediated complete depletion of PPARδ also showed an altered OPG/RANKL ratio and an increased osteoclast differentiation^14^, the current Runx-2-meidated osteoblast-specific deletion of this nuclear receptor primarily resulted in osteoblast-intrinsic effects and reduced mineralization without increasing osteoclast differentiation, suggesting predominant effects of PPARδ on osteoblast metabolism, differentiation and mineralization. The exact reason for this discrepancy is unclear, but might be due an additional PPARδ-mediated control of the OPG/RANKL system in cells others than osteoblasts.

PPARδ belongs to the nuclear receptor superfamily of transcription factors. Its close family member PPARγ acts as key factor during adipocyte differentiation. Although both PPARγ and PPARδ sense a repertoire of lipophilic compounds and subsequently act as ligand-activated transcription factors, they dictate partially opposing cellular pathways such as fatty acid synthesis and fatty acid oxidation. Moreover, PPARγ and PPARδ are differentially expressed in distinct cell types such as adipocytes and osteoblasts. Ligand-induced activation of these different PPAR family members accordingly exerts contrasting effects on both systemic energy and bone metabolism^15^. These findings highlight the importance of such metabolic sensors during cellular fate decision processes and additionally provide an example where cellular metabolism and cellular differentiation of osteoblasts and other mesenchymal cell types are mutually interlinked.

## Methods

The authors confirm that all methods were carried out in accordance with relevant guidelines and regulations.

### Animals

All animal experiments were approved and carried out in accordance with relevant guidelines and regulations by the government of Unterfranken, Peterplatz 1, 97070 Würzburg, Germany. Mice were maintained at the specific pathogen-free animal care facility (FPZ) of the University of Erlangen-Nuremberg and housed in a room at 23 ± 2 °C, with 50 ± 10% humidity and a 12-hour light/dark cycle (lights on from 08:00 a.m. to 08:00 p.m.). All mice were allowed free access to water and regular rodent chow. Mice with floxed PPARdelta allele exon (PPAR^fl/fl^) were kindly supplied from Béatrice Desvergne, Center of Integrative Genomics, Faculty of Biology and Medicine, University of Lausanne, Switzerland^14^. Runx2-Cre^tg/+^ mice were a gift from Jan Tuckermann, Institute of Comparative Molecular Endocrinology, University of Ulm, Germany.

### Bone analysis

We measured the structure of tibial and vertebral bones with a SCANCO Medical μCT 40 scanner to produce the images. Results were analyzed with SCANCO evaluation software for segmentation, three-dimensional morphometric analysis, density and distance parameters (SCANCO Medical AG). We performed histomorphometric analysis with the OsteoMeasure Analysis System (Osteo-Metrics) to determine osteoclast and osteoblast parameters as previously described^18^.

### Cell culture

Isolation of calvarial osteoblasts was previously described^18^. For osteoblast monocultures, we differentiated freshly isolated calvarial osteoblast precursors in the presence of 5 mM ß-glycerolphosphate (CALBIOCHEM #35675) and 100 mg/ml ascorbic acid (Sigma-Aldrich #A0278). MSCs were isolated and fully characterized from PPARδ wildtype and PPARδ-deficient mice bone-marrow as described previously^19^ confirming the mesenchymal stem cell minimal criteria^20^. We plated both MSC and osteoblast cell suspensions (0.5 × 10^6^ cells cm^−2^) in α-MEM (GIBCO® by life technologies #32571-028) supplemented with 10% FBS (Biowest #S1580), 100 U/ml penicillin and 10 mg/ml streptomycin (PAN Biotech GmbH #P06-07100), at 37 °C in humidified atmosphere containing 5% CO2 in air. Culture media were changed every 2 days. At subconfluence, we plated cells at a density of 5 000 cells cm^−2^. MSC were used between passages 7 and 12.

### Immunohistochemistry

PPARdelta immunohistochemical expression was evaluated MSC at day 7 after induction of osteoblast differentiation We used polyclonal anti-PPARβ from rabbit (Santa Cruz Biotechnology, #sc-7197) in a dilution of 1:100 in 2% BSA/PBS as previously described^21^. Slides were viewed under the fluorescence microscope ECLIPSE Ni-Series microscope, Nikon, USA.

### Mineralization assay

To boost mineralization osteoblasts were cultured in a medium containing 10 ng/ml recombinant mouse Wnt3a protein (R&D #NP_149122), ß-glycerolphosphate (CALBIOCHEM #35675) and ascorbic acid (Sigma-Aldrich #A0278).

Cells were stimulated with Tigecycline 30 µM. At indicated time points, cells were briefly washed with PBS and then fixed with 95% Ethanol for 30 minutes at room temperature. After briefly washing mineralized spots were dyed with 2% Alizarin Red S (Merck #A5533) in H_2_O (pH adjusted between 4 and 4,3) for 3 to 5 minutes. Then wells were washed with H_2_O for 5 to 8? times. Stained plates were dried and pictures were taken using the HP Scanjet G4050 and HP Scansoftware. Calcification areas were quantified with help of adobe photoshop CS6 software.

### Real-time PCR analysis

We isolated total RNA from cells by using TRIZOL reagent (Invitrogen). Concentrations were measured via NanoDrop analysis. 1 µg of isolated RNA was used for the first-strand complementary DNA synthesis (Amersham Biosciences), which was then used for SYBR Green–based quantitative RT-PCR as described previously^22^. Experiments were performed in triplicate. Normalized gene expression values for each sample were calculated as the ratio of expression of messenger RNA (mRNA) for the gene of interest to the expression of mRNA for β-actin using QuantStudio™ 6 and 7 Flex Real-Time PCR System Software Base v1.x. The following real-time PCR primer sequences were used: *ALP, 5*′*-*CACGCGATGCAACACCACTCAGG-3′ (sense) and 5′-GCATGTCCCCGGGCTCAAAGA-3′ (antisense); *CPT1, 5*′*-*CCAGGCTACAGTGGGACATT-3′ (sense) and 5′-GAACTTGCCCATGTCCTTGT-3′ (antisense); *DRP1, 5*′*-*CTG ACG CTT GTG GAT TTA CC-3′ (sense) and 5′-CCC TTC CCA TCA ATA CAT CC-3′ (antisense); *Glut1, 5*′*-*TCAACACGGCCTTCACTG-3′ (sense) and 5′-CACGATGCTCAGATAGGACATC-3′ (antisense); *Glut3, 5*′*-*TTCTGGTCGGAATGCTCTTC-3′ (sense) and 5′-AATGTCCTCGAAAGTCCTGC-3′ (antisense); *OCN, 5*′*-*ACCCTGGCTGCGCTCTGTCTCT-3′ (sense) and 5′-GATGCGTTTGTAGGCGGTCTTCA-3′ (antisense); *Osx*, 5′-GGAGGCACAAAGAAGCCATACGC-3′ (sense) and 5′-TGCAGGAGAGAGGAGTCCATTG-3′ (antisense); *PDK4, 5*′*-*CCT TCA CAC CTT CAC CAC AT-3′ (sense) and 5′-AAA GAG GCG GTC AGT AAT CC-3′ (antisense); *PGC1alpha, 5*′*-*TATGGAGTGACATAGAGTGTGCT-3′ (sense) and 5′-CCACTTCAATCCACCCAGAAAG-3′ (antisense); *Ppara*, 5′-GGACCTTCGGCAGCTGGT-3′ (sense) and 5′-TCGGACTCGGTCTTCTTGATG-3′ (antisense); *Ppard*, 5′-CTGAAGGGAAGGGGGTAGAG-3′ (sense) and 5′-CAGTCTGGATGCTGCTACA-3′ (antisense); *Pparg*, 5′-CATTCTGGCCCACCAACTTC-3′ (sense) and 5′-TCAAAGGAATGCGAGTGGTCTT-3′ (antisense); *Runx2*, 5′-GACGAGGCAAGAGTTTCACC-3′ (sense) and 5′-GGACCGTCCACTGTCACTTT-3′ (antisense); *TFAM, 5*′*-*GATGGCGCTGTTCCGG-3′ (sense) and 5′-TGGATAGCTACCCATGCTGGA-3′ (antisense) and *UCP3, 5*′*-*CCTACGACATCATCAAGGAGAAGTT-3′ (sense) and 5′-TCCAAAGGCAGAGACAAAGTGA-3′ (antisense). Normalized gene expression values for each sample were calculated as the ratio of expression of mRNA of the gene of interest to the expression of mRNA for β-actin.

### Metabolic analysis

The cells’ bioenergetics were assessed using an XFe96 Extracellular Flux Analyzer (Seahorse Bioscience, North Billerica, MA) as well as corresponding kits (Agilent, Santa Clara, California, USA). Cells were seeded in an optimized concentration of 15 000 cells per well. One hour before performance of seahorse experiment, cells were incubated at 37 °C in a CO_2_-free atmosphere. XF Mitochondrial Stress Test Kits (Agilent, #103015) and XF Glycolysis Stress Test Kits (Agilent, #103020) were utilized according to the user guide (Seahorse Bioscience) and as described before^23^. For the Mitochondrial Stress Test, basal oxygen consumption rate (OCR) (an indicator for mitochondrial respiration) and extracellular acidification rate (ECAR) (an indicator for lactic acid production or glycolysis) were analyzed. Next, OCR and ECAR responses toward the application of oligomycin (1 µM), FCCP (2.5 µM), and the combination of antimycin (3 µM) and rotenone (3 µM) were evaluated. For Glycolysis Stress Test, OCR and ECAR were detected. Then, OCR and ECAR responses toward the application of Glucose (100 mM), Oligomycin (100 µM) and 2-DG (500 mM) were evaluated.

### Chromatin immunoprecipitation (ChIP) assays

To verify the binding of PPAR*d* on selected promoters, ChIP assays were performed according to manufacturer instructions using the ChIP-IT® Express Chromatin Immunoprecipitation Kit (#53008, Active Motif, CA, USA). 25 μg of sonicated chromatin extract were incubated with 3.4 µg specific antibodies against PPARdelta (#ab178866, Abcam, Cambridge, MA, USA) or normal rabbit IgG antibodies (#sc-2027, Santa Cruz Biotechnology, CA, USA). After purification of DNA using Chromatin IP DNA Purification Kit (Active Motif, #58002) qPCR reactions were carried out on specific genomic regions using SYBR Green (Bio-Rad, USA). The resulting signals were normalized for primer efficiency by carrying out qPCR for each primer pair using the genomic input DNA. For positive control ChIP H3K36me3 antibody was used.

The following primers were used in these ChIP assays: PPRE on UCP3 promoter 5′-TGTGTTGCAGACAGAAGATGG-3′ (sense) and 5′-ACCCCTCGTTTTACCAAAAGCAG-3′ (antisense); PPRE on CTP1 promoter 5′-TGCTAGAGATCAGTCGGTGAG-3′ (sense) and 5′-CTTGAACTCAGAAATCCGCCTG-3′ (antisense) and PPRE on PDK4 promoter 5′-GTCCACTAAACAGCAGAGCAC-3′ (sense) and 5′-GAAAGTCATAGATGACGGTGG-3′ (antisense).

### Statistical analyses

All data are presented as mean ± SEM. Tests for statistical significance were performed with Student’s *t* test using GraphPad Prism Version 5 (GraphPad Prism Software Inc. La Jolla, California, USA). P < 0.05 was considered as significant.

## Supplementary information

Supplementary information.
