# Supplementary material for: PPARδ-mediated mitochondrial rewiring of osteoblasts determines bone mass
Source: Sci Rep. 2020 May 21;10:8428. doi: 10.1038/s41598-020-65305-5 (PMC7242479; doi:10.1038/s41598-020-65305-5)
Supplement: Supplementary file 1 — Supplementary information. [file 41598_2020_65305_MOESM1_ESM.pdf]

PPARδ-mediated mitochondrial rewiring of osteoblasts determines bone mass

Dorothea I. H. Müller, Cornelia Stoll, Katrin Palumbo-Zerr, Christina Böhm, Brenda Krishnacoumar, Natacha Ipseiz, Jule Taubmann, Max Zimmermann, Martin Böttcher, Dimitrios Mougiakakos, Jan Tuckermann, Farida Djouad, Georg Schett, Carina Scholtysek, Gerhard Krönke

Supplementary Figure 1

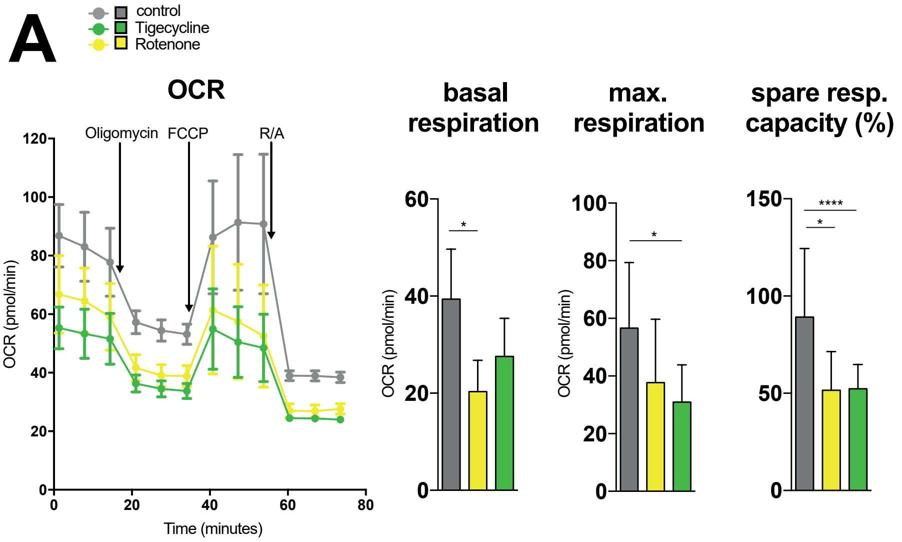

Supplementary Figure 1|
Metabolism of calvarial osteoblasts was assessed using an extracellular flux (XF) analyzer.
(A) OCR, basal and maximal respiration rate, %spare respiratory capacity measured in freshly isolated calvarial osteoblasts cultured in differentiaton medium (grey) conditionally supplemented with Tigecycline 30 µM (green) and Rotenone 0,02 µM (yellow) for 24 hours.

Statistics
Figure 1A: shown is the mean of biological replicates and two-tailed unpaired Student's t test, n = 14-19
